# Efficacy of Kegan Liyan oral liquid vs. Lianhuaqingwen capsules for patients with mild COVID-19: a double-blinded, randomized, controlled, non-inferiority trial

**DOI:** 10.3389/fmed.2025.1531370

**Published:** 2025-02-26

**Authors:** Yuewei Li, Yihe Chi, Mengting Zhu, Feiting Fan, Zhongyang Deng, Jingmin Xiao, Shaohan Jin, Luoqi Lin, Xiaochun Chen, Ruhong Xu, Long Fan, Xuhua Yu, Ziyao Liang, Jingyu Quan, Shangzhao Li, Xinying Peng, Yuanbin Chen, Lin Lin, Lei Wu

**Affiliations:** ^1^State Key Laboratory of Traditional Chinese Medicine Syndrome/The Second Clinical College of Guangzhou University of Chinese Medicine, Guangzhou, China; ^2^The Second Affiliated Hospital of Guangzhou University of Chinese Medicine/Guangdong Provincial Hospital of Chinese Medicine, Guangzhou, China; ^3^The Ninth People's Hospital of Dongguan, Dongguan, China; ^4^Guangdong-Hong Kong-Macau Joint Lab on Chinese Medicine and Immune Disease Research, Guangzhou University of Chinese Medicine, Guangzhou, China

**Keywords:** coronavirus disease 2019, Kegan Liyan oral liquid, Lianhuaqingwen capsules, non-inferiority, randomized controlled trial

## Abstract

**Background:**

Traditional Chinese medicine has been used for Coronavirus disease 2019 (COVID-19) as a therapeutic option. Lianhuaqingwen capsules (LHQW) are well-recognized for their efficacy, while Kegan Liyan oral liquid (KGLY), widely used for influenza treatment, has emerged as a promising candidate for COVID-19 therapy. This trial aims to assess whether KGLY is non-inferior to LHQW in treating mild COVID-19.

**Methods:**

A total of 127 participants (63 in KGLY group and 64 in LHQW group) were randomly allocated to receive either KGLY therapy or LHQW therapy in a 1:1 ratio. The treatment was given for 7 days, and the follow-up period was 3 days.

**Outcome measures:**

The primary outcome was symptom remission at day 10. Secondary outcomes included symptom recovery, time to symptom remission, recovery rates and time to recovery of selected symptoms, change in visual analog scale score for selected symptoms, area under the curve of the visual analog scale score for sore throat, negative conversion of the SARS-CoV-2 infection, having a positive test result after negative conversion, and incidence of pneumonia.

**Results:**

Full analysis set analysis showed that the symptom remission rate at day 10 was 60.7% with KGLY and 58.3% in LHQW (difference + 2.3 p.p., lower limit of 95% confidence interval − 14.8 p.p.), indicating non-inferiority. There were no significant differences between the groups for any secondary outcome. The occurrence of adverse events did not differ between the groups and no severe adverse events were documented in either group.

**Conclusion:**

Based on the study results, this trial proved that KGLY was non-inferior to LHQW for mild COVID-19, providing a promising option for COVID-19 treatment.

**Clinical trial registration:**

https://www.chictr.org.cn/showproj.html?proj=166372, Identifier, [ChiCTR2200059105].

## Introduction

Coronavirus disease 2019 (COVID-19), caused by the severe acute respiratory syndrome coronavirus 2 (SARS-CoV-2), has rapidly spread over the world since the first cases that were observed in December 2019. In 2022, the Omicron variant rapidly surpassed other circulating strains around the world. Mild influenza-like symptoms including fever, sore throat, cough, fatigue and myalgia were reported to be the prevalent symptoms of Omicron (1). As of May 2024, the number of COVID-19 cases keeps on rising, with 775 million reported cases and 7.0 million deaths (2). Owing to the high transmissibility and the possibility of complications and lingering after-effects, the demand for timely interventions was prominent.

Traditional Chinese medicine (TCM) has been applied to treat pandemics for thousands of years and has shown potential efficacy against COVID-19 (3). For example, Lianhuaqingwen capsules (LHQW), a widely used Chinese herbal product, have been shown to significantly mitigate symptoms and accelerate clinical recovery in COVID-19 patients (4–8), and are recommended in the Chinese guideline for the diagnosis and treatment of COVID-19 (Trial 10th edition, 2023) (9). However, to date, high-quality evidence-based evaluations of TCM treatments are still few, the scarcity is particularly notable in the context of the need for more stringent controlled trials to substantiate the claims of TCM’s efficacy. This gap hinders the broader recognition and integration of TCM into mainstream COVID-19 treatment strategies. Hence, the search for more TCM therapeutic options against COVID-19 remains an important research goal.

Kegan Liyan oral liquid (KGLY), acting by exerting heat-clearing and dampness-resolving effects, has been used extensively for the treatment of influenza. Multiple clinical trials have demonstrated that KGLY exerts beneficial effects in patients with various respiratory diseases including influenza, chronic pharyngitis, acute upper respiratory tract infection, and acute suppurative tonsillitis (10–12). Based on this evidence, KGLY could be a promising candidate for COVID-19 treatment. This therapy is also recommended by the expert consensus on Chinese patent medicine and TCM treatment for COVID-19 in Guangdong Province (13, 14).

However, the efficacy of KGLY against COVID-19 has not been proven yet. Therefore, in this study, we conducted a double-blinded, randomized, controlled, non-inferiority trial design to determine the efficacy of KGLY using the well-established LHQW as a positive control, to provide objective and scientific evidence for the use of KGLY for COVID-19.

## Methods

### Study design

A double-blinded, double-dummy, randomized, controlled, non-inferiority clinical trial was conducted at two public hospitals in China (The Ninth People’s Hospital of Dongguan, and the Guangdong Provincial Hospital of Chinese Medicine). Before commencing recruitment, the participating centers had acquired ethics approval. The trial was registered in April 2022 (Chinese Clinical Trial Registry: No.ChiCTR2200059105) and was conducted strictly according to the Consolidated Standards of Reporting Trials (CONSORT) Statement. All participants signed an informed consent.

### Participants

We recruited all patients who were treated in the two participating hospitals between July 2022 and January 2023. Patients fulfilling all of the following criteria were eligible: (1) aged 18 to 75 years; (2) having a laboratory-confirmed diagnosis of mild COVID-19 in accordance with the guideline on diagnosis and treatment of COVID-19 (Trial 9th edition) (15); (3) meeting the diagnostic criteria of the *Dampness-heat in the Lung* TCM syndrome (15); (4) body temperature ≤ 38.5°C since the onset of illness; (5) having symptoms (either fever, sore throat, cough, myalgia, or fatigue); and (6) the patient voluntarily signed the informed consent.

We excluded patients who met at least one of the following criteria: (1) having a chronic respiratory disease such as chronic obstructive pulmonary disease, bronchial asthma, bronchiectasis, active pulmonary tuberculosis, lung malignant tumors, or interstitial lung disease; (2) having severe comorbidities of the cardio-cerebrovascular, renal, hepatic, or blood system, malignant tumors, or other serious primary diseases, or alanine aminotransferase, aspartate aminotransferase or serum creatinine levels that exceeded 1.5 times the upper limit of normal; (3) due to neurological and mental disease (based on medical history), unable to cooperate; (4) having allergies or hypersensitivity for any research drug component; (5) having peptic ulcer or digestive hemorrhage; (6) being pregnant, lactating, or preparing for pregnancy; or (7) having received any experimental treatment within the previous 3 months.

### Randomization, allocation concealment and blinding

A verified interactive web response method was used for randomization by an independent third-party unit, the Key Unit of Methodology in Clinical Research (KUMCR) of Guangdong Provincial Hospital of Chinese Medicine. SAS version 9.4 (SAS Institute Inc., Cary, United States) was used for all randomization processes. Patients were randomly assigned to KGLY or LHQW arms in a 1:1 ratio using a randomized block design.

KUMCR staff had access to the randomization list and blinding codes, which were kept strictly confidential. As a result, during the trial, all participants and outcome evaluators were not aware of the treatment allocation.

### Interventions

Enrolled patients were randomly split into the KGLY and LHQW groups at a ratio of 1:1. In the KGLY group, 20 mL of KGLY and four capsules of LHQW-like placebo were administered three times a day orally, for 7 days. In the LHQW group, patients were given 20 mL of KGLY-like placebo and four capsules of LHQW three times a day orally for 7 days. Patients were instructed to bring the remaining medication or medicine box back after treatment for medication counting. Patients who recovered were asked to discontinue treatment ahead of time.

KGLY (National Drug Approval Z10970100), LHQW (National Drug Approval Z20040063) and the placebo were all provided by Wanglaoji Pharmaceutical Co., Ltd. (Guangzhou, China). Drugs were all examined in advance through the standard procedure, which met the requirements of Chinese Pharmacopoeia (2020 edition). Tables 1–3 display the ingredients of KGLY, LHQW and the placebo. In terms of appearance, taste and smell, the placebo matched the research products.

**Table 1:** Composition of the KGLY formula.

Name of the component (herb)						
Chinese name	Latin name^a^	English name	Processing method	Medicinal part	Concentration (g per 1,000 mL of KGLY)	Category of medicine^b^
Jin Yin Hua	*Lonicera japonica Thunb.*	Flos Lonicerae Japonicae	/	Flower bud or opening flower	72	Chief
Huang Qin	*Scutellaria baicalensis Georgi*	Radix Scutellariae	Sliced	Root	72	Chief
Jing Jie	*Schizonepeta tenuifolia Briq.*	Herba Schizonepetae	/	Aerial part	72	Deputy
Zhi Zi	*Gardenia jasminoides Ellis*	Fructus Gardeniae	/	Fruit	72	Deputy
Lian Qiao	*Forsythia suspensa (Thunb.) Vahl*	Fructus Forsythiae	/	Fruit	72	Deputy
Xuan Shen	*Scrophularia ningpoensis Hemsl.*	Radix Scrophulariae	Sliced	Root	72	Deputy
Jiang Can	*Bombyx mori Linnaeus.*	Bombyx Batryticatus	Stir-baked with ginger juice	Body	43	Deputy
Di Huang	*Rehmannia glutinosa Libosch.*	Radix Rehmanniae	Sliced	Tuberous root	108	Assistant
She Gan	*Belamcanda chinensis (L.) DC.*	Rhizoma Belamcandae	Sliced	Rhizome	22	Assistant
Jie Geng	*Platycodon grandiflorum (Jacq.) A.DC.*	Radix Platycodonis	Sliced	Root	43	Assistant
Bo He	*Mentha haplocalyx Briq.*	Herba Menthae	/	Aerial part	43	Assistant
Chan Tui	*Cryptotym panapustulata Fabricius*	Periostracum Cicadae	/	Periostracum	43	Assistant
Fang Feng	*Saposhnikovia divaricate (Turcz.) Schischk.*	Radix Saposhnikoviae	Sliced	Root	43	Assistant
Gan Cao	*Glycyrrhiza uralensis Fisch.*	Radix Et Rhizoma Glycyrrhizae	Sliced	Root and rhizome	22	Envoy

a Latin names above have been verified from http://www.theplantlist.org, https://wfoplantlist.org/ and the Chinese Pharmacopeia (2020 edition).

b Chinese herbs are typically prescribed in formulas that include ‘chief’ medicines, which provide the most potent therapeutic action; ‘deputy’ medicines, which assist ‘chief’ medicines in their therapeutic actions; ‘assistant’ medicines, which aid ‘deputy’ medicines in treating other disease-related symptoms; and ‘envoy’ medicines, which may help by guiding the treatment action and balancing the effect of other drugs.

KGLY, Kegan Liyan oral liquid.

**Table 2:** Composition of the LHQW formula.

Name of the component (herb)						
Chinese name	Latin name^a^	English name	Processing method	Medicinal part	Concentration (g per 1,000 capsules of LHQW)	Category of medicine^b^
Lian Qiao	*Forsythia suspensa (Thunb.)Vahl*	Fructus Forsythiae	/	Fruit	255	Chief
Jin Yin Hua	*Lonicera japonica Thunb.*	Flos Lonicerae Japonicae	/	Flower bud or opening flower	255	Deputy
Ma Huang	*Ephedra sinica Stapf*	Herba Ephedrae	Sliced and honey-fried	Herbaceous stem	85	Deputy
Ban Lan Gen	*Isatis indigotica Fort.*	Radix Isatidis	Sliced	Root	255	Assistant
Guang Huo Xiang	*Pogostemon cablin (Blanco) Benth.*	Herba Pogostemonis	Sliced	Aerial part	85	Assistant
Da Huang	*Rheum palmatum L.*	Radix Et Rhizoma Rhei	Sliced	Root and rhizome	51	Assistant
Mian Ma Guan Zhong	*Dryopteris crassirhizoma Nakai*	Rhizoma Dryopteridis Crassirhizomatis	Sliced	Rhizome and frond bases	255	Assistant
Hong Jing Tian	*Rhodiola crenulata* (*Hook. f. et Thoms.*) *H. Ohba*	Radix Et Rhizoma Rhodiolae Crenulatae	Sliced	Root and rhizome	85	Assistant
Yu Xing Cao	*Houttuynia cordata Thunb.*	Herba Houttuyniae	Sliced	Aerial part	255	Assistant
Ku Xing Ren	*Prunus sibirica L.*	Semen Armeniacae Amarum	Shattered	Ripe seed	85	Assistant
Shi Gao	/	Gypsum Fibrosum	Shattered	CaSO_4_·2H_2_O	255	Assistant
Bo He Nao	*Mentha haplocalyx Briq.*	Mentholum	Distilled and recrystallization	C_10_H_20_O	7.5	Assistant
Gan Cao	*Glycyrrhiza uralensis Fisch.*	Radix Et Rhizoma Glycyrrhizae	Sliced	Root and rhizome	85	Envoy

a Latin names above have been verified from http://www.theplantlist.org, https://wfoplantlist.org/ and the Chinese Pharmacopeia (2020 edition).

b Chinese herbs are typically prescribed in formulas that include ‘chief’ medicines, which provide the most potent therapeutic action; ‘deputy’ medicines, which assist ‘chief’ medicines in their therapeutic actions; ‘assistant’ medicines, which aid ‘deputy’ medicines in treating other disease-related symptoms; and ‘envoy’ medicines, which may help by guiding the treatment action and balancing the effect of other drugs.

LHQW, Lianhuaqingwen capsules.

**Table 3:** Composition of the placebo alternatives for KGLY and LHQW.

	Ingredient	Concentration (g per 1,000 mL solvent; solvent: water)
Composition of the placebo for KGLY	Polysorbate 80	1 g
	Caramel color	2.5 g
	Bitterant	1.5 g
	Sodium benzoate	3 g
Composition of the placebo for LHQW	Ingredient	Concentration (g per 1,000 capsules)
	Sodium carboxymethyl cellulose	4 g
	Caramel color	56 g
	Corn starch	290 g

KGLY, Kegan Liyan oral liquid; LHQW, Lianhuaqingwen capsules.

The reliever drug, paracetamol tablet, was manufactured by Sinopharm Shantou Jinshi Pharmaceutical Co., Ltd. (Shantou, China, National Drug Approval H44021051). Patients were allowed to take the reliever medication when suffering from pain or having subaxillary temperature ≥ 38.5°C.

The use of additional medications for COVID-19, such as antitussive, antiviral and phlegm-resolving drugs, and Chinese medicine for influenza and common cold, was restricted during the trial. Subjects were excluded from the trial if discontinuing these medications was deemed clinically harmful or if patients were unwilling to do so. Except for the medications indicated above, therapy for underlying conditions such as hypertension could remain unaltered.

### Efficacy and safety assessment

The data were obtained through a primary research method. Investigator trainings were conducted prior to the trial according to developed standard operating procedures. Following enrollment, participants were instructed to note on their diary records each day their body temperature, symptom visual analog scale (VAS) score and the total amount and frequency of paracetamol tablet consumption. The VAS was a 10 cm visual horizontal line, where patients scored the severity of their symptoms (sore throat, cough, myalgia and fatigue) from 0 to 10 (0 = no symptom, 10 = worst possible symptom). During the study period, diary records were regularly verified by investigators to ensure that data were filled in correctly. For antiviral efficacy, throat swab samples were collected for SARS-CoV-2 RNA testing at baseline, day 3, day 7, and day 10. In addition, routine blood, renal and hepatic function testing was performed at baseline as well as day 7. Throughout the study, adverse events and concomitant drugs were monitored and documented. We considered the following adverse events: (1) new onset of a disease or symptoms or signs, or a clinically significant progression and deterioration of concomitant diseases; or (2) adverse events related to clinical laboratory tests and other examinations. The severity of adverse events was divided into three categories-mild, moderate, and severe-according to the Common Terminology Criteria for Adverse Events (16).

### Outcome measures

The primary outcome was symptom remission at 10 days since treatment initiation. Remission of symptoms was defined as having no fever, as well as all of the VAS scores of sore throat, cough, myalgia and fatigue having stayed between 0 and 1 cm for more than 48 h (VAS was a scale ranged from 0 to 10 cm). Fever was defined as a subaxillary temperature ≥ 37.3°C. The magnitude of sore throat, cough, myalgia and fatigue was reported by patients in their diaries using VAS scores.

Secondary outcomes included: (1) symptom recovery (recovery of symptoms was defined as having no fever, as well as all of the VAS scores of sore throat, cough, myalgia and fatigue having stayed at 0 cm for more than 48 h); (2) time to symptom remission, defined as the time span between the initial intervention and symptom remission; (3) recovery of selected symptoms (fever, sore throat, cough, myalgia and fatigue); (4) time to recovery of these symptoms; (5) change in VAS score of these symptoms; (6) the area under the curve (AUC) of the VAS score for sore throat; (7) SARS-CoV-2 RNA negative conversion; (8) a positive SARS-CoV-2 RNA test result after negative conversion; and (9) incidence of pneumonia.

### Sample size calculation

The sample size was calculated using the PASS 15 software. In line with a prior study (4), the symptom recovery rate of LHQW for COVID-19 at day 10 was expected to be 78% and that in the control group 45%, with a 95% CI 22 to 43%. For the reason that rate of symptom remission must be higher than that of symptom recovery in the same period, we assumed 89% of participants achieve the condition of symptom remission at day 10. The non-inferiority margin was set at −17 p.p., the significance threshold at 0.025, and power at 80% (*β* = 0.2), implying that each arm needed at least 54 cases to confirm non-inferiority. With a 10% dropout rate, this corresponds to a sample size of 120 patients (60 subjects per group).

### Statistical analysis

All data were independently entered into EpiData software by two personnel and cross-checked. Differences were carefully corrected by consulting the original report forms to minimize input errors. For statistical analysis, IBM SPSS software (version 26.0) was used. To impute missing data, the last observation carried forward (LOCF) approach was utilized.

We conducted the analyses in three datasets, including the full analysis set (FAS), the per protocol set (PPS) and the safety set (SS). We presented continuous variables using means (with standard deviation, SD) or median (with interquartile range, IQR) and categorical variables using frequencies (n) and percentages (%). The primary outcome (symptom remission rate) of KGLY and LHQW arms was compared by Chi-squared test or Fisher’s exact test. Non-inferiority test was employed for the primary endpoint. The outcomes of time to symptom remission and recovery of selected symptoms were analyzed using log-rank test and Cox regression. The *t*-test (for normally or almost normally distributed data) or the Wilcoxon rank-sum test (for severely skewed variables) were used to compare continuous outcomes (AUC of the VAS score for sore throat and VAS score for sore throat, cough, myalgia and fatigue) between arms. Binary outcomes (symptom recovery, recovery of selected symptoms, SARS-CoV-2 RNA negative conversion, positive test result after negative conversion, and incidence of pneumonia) were analyzed using the Chi-square test or Fisher’s exact test. For paired samples, paired *t*-test was used for normally or almost normally distributed data, or the Wilcoxon signed-rank test for severely skewed data. A one-tailed test was applied only for the non-inferiority test of the primary outcome, and all other tests were two-tailed. Statistical significance was set at 0.05.

## Results

### Randomization and baseline characteristics

**Figure 1:**
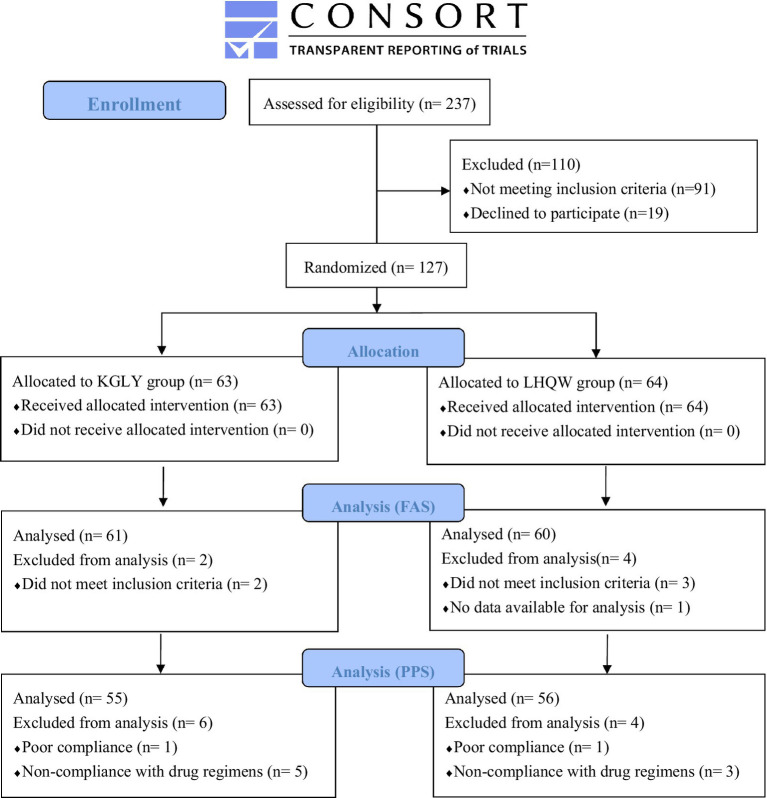
Consolidated standards of reporting trials (CONSORT) flow diagram. KGLY, Kegan Liyan oral liquid; LHQW, Lianhuaqingwen capsules; FAS, full analysis set; PPS, per protocol set.

From July 2022 to January 2023, 127 participants were randomly allocated to KGLY group (*n* = 63) or LHQW group (*n* = 64). Finally, 121 participants (61 in KGLY arm and 60 in LHQW arm) qualified for FAS; Six participants (2 in KGLY and 4 in LHQW groups) were excluded from the FAS due to not meeting the inclusion criteria or having no available data for analysis. Furthermore, 111 participants (55 in KGLY and 56 in LHQW groups) qualified for PPS, with 16 of the allocated participants (8 in each group) excluded (Figure 1). During the study period, seven participants dropped out (4 in KGLY and 3 in LHQW groups, *p* = 0.68). Baseline demographic and clinical characteristics were in balance between the groups. The median age was 34 years, and 72 (59.5%) participants were female. The median time since the onset of illness at baseline was 3 days in both groups (IQR 2–4). Cough (*n* = 113, 93.4%) and sore throat (*n* = 112, 92.6%) were the most common symptoms (Table 4).

**Table 4:** Baseline demographic and clinical characteristics in the KGLY and LHQW arms.

Characteristics	KGLY (*n* = 61)	LHQW (*n* = 60)	*p* value
Male, n (%)	29 (47.5)	20 (33.3)	0.11
Age in years, median (interquartile range)	30 (27, 42)	30 (25, 44)	0.82
Body mass index, median (interquartile range)	21.5 (19.6, 24.3)	22.1 (20.0, 24.3)	0.70
Outpatient, n(%)	59 (96.7)	58 (96.7)	0.99
History of other diseases, n (%)	12 (19.7)	12 (20.0)	0.96
History of drug allergy, n (%)	9 (14.8)	10 (16.7)	0.77
COVID-19 vaccination, n (%)	60 (98.4)	59 (98.3)	1.00
Time since the onset of illness in days, median (interquartile range)	3 (2, 4)	3 (2, 4)	0.61
Time since the confirmation of SARS-CoV-2 infection in days, median (interquartile range)	2 (0, 3)	2 (1, 3)	0.87
The highest body temperature within 24 h (°C), mean (SD)	37.8 (0.8)	37.9 (0.6)	0.43
Body temperature at baseline (°C), mean (SD)	37.0 (0.7)	37.0 (0.6)	0.96
Fever^a^, n (%)	41 (67.2)	45 (75.0)	0.34
Sore throat, n (%)	57 (93.4)	55 (91.7)	0.98
Cough, n (%)	59 (96.7)	54 (90.0)	0.26
Myalgia, n (%)	41 (67.2)	45 (75.0)	0.34
Fatigue, n (%)	52 (85.2)	48 (80.0)	0.45
VAS score for sore throat severity, mean (SD)	4.8 (2.4)	4.6 (2.7)	0.59
VAS score for cough severity, mean (SD)	4.5 (2.4)	4.3 (2.9)	0.74
VAS score for myalgia severity, mean (SD)	3.1 (3.0)	2.9 (2.8)	0.64
VAS score for fatigue severity, mean (SD)	3.6 (2.7)	3.6 (2.9)	0.96

KGLY, Kegan Liyan oral liquid; LHQW, Lianhuaqingwen capsules; VAS, visual analog scale; SD, standard deviation.

a Fever was defined as a subaxillary temperature ≥ 37.3°C.

### Primary outcome

**Figure 2:**
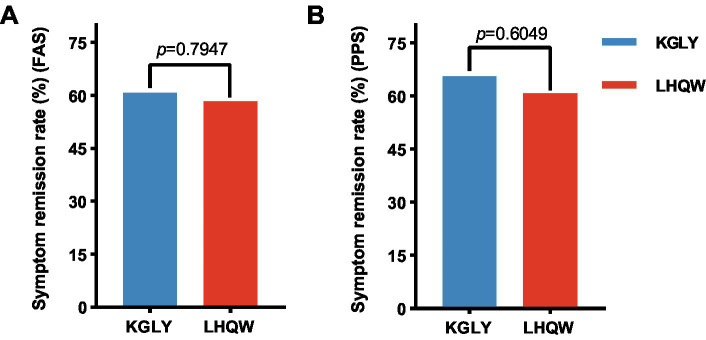
Symptom remission rate at day 10 in the KGLY and LHQW groups. Remission of symptoms was defined as having no fever, as well as all visual analog scale scores (sore throat, cough, myalgia and fatigue) staying between 0 and 1 cm for more than 48 h. KGLY, Kegan Liyan oral liquid; LHQW, Lianhuaqingwen capsules.

**Figure 3:**
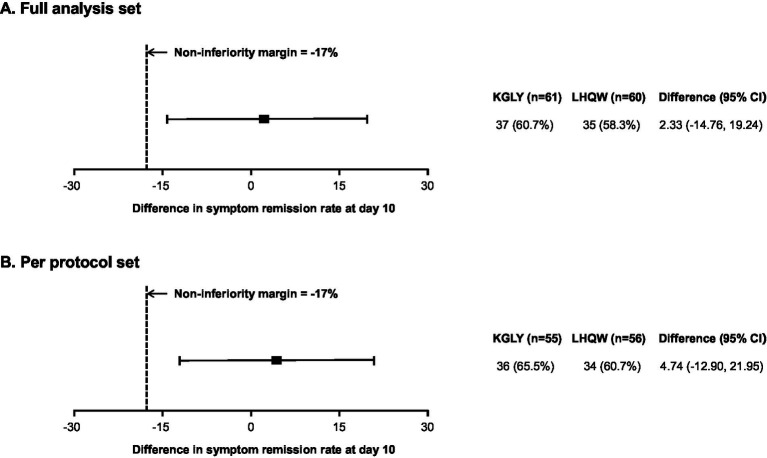
Results of the non-inferiority test for KGLY vs. LHQW in symptom remission rate at day 10.Non-inferiority would be shown if the lower limit of the 95% CI of the risk difference was higher than the −17 p.p. non-inferiority margin. KGLY, Kegan Liyan oral liquid; LHQW, Lianhuaqingwen capsules; CI, confidence interval; p.p., percentage point.

In the FAS analysis, at day 10, symptom remission rate was 60.7% in the KGLY arm and 58.3% in the LHQW arm: the difference was +2.3 p.p. and lower limit of the 95% CI -14.8 p.p., indicating non-inferiority (Figures 2A, 3A). In the PPS analysis, symptom remission rate was 65.5% in KGLY and 60.7% in LHQW groups, also validating the non-inferiority of KGLY (difference + 4.7 p.p., lower limit of the 95% CI -12.9 p.p.; Figures 2B, 3B).

### Secondary outcomes

No significant differences within groups were observed in symptom recovery rate between KGLY and LHQW arms at day 3 (0.0% vs. 1.7%, *p* = 0.99) and day 7 (11.5% vs. 11.7%, *p* = 0.97). At day 10, 23.0% of the patients in KGLY group had achieved symptom recovery, not statistically different comparing with that in LHQW group (26.7%; difference − 3.7 p.p., *p* = 0.63; Table 5). The median time to symptom remission in KGLY and LHQW group were both 10 days, and and there was no statistical difference between groups (*p* = 0.62; Table 6).

**Table 5:** Symptom recovery rate in the KGLY and LHQW arms (FAS).

	Day 3	Day 7	Day 10
Symptom recovery rate^a^	KGLY: *n* = 61; LHQW: *n* = 60		
KGLY, n (%)	0 (0.0)	7 (11.5)	14 (23.0)
LHQW, n (%)	1 (1.7)	7 (11.7)	16 (26.7)
RR (95% CI)	/	0.99 (0.57 to 1.73)	0.91 (0.61 to 1.35)
*p* value	0.99	0.97	0.63

a Symptom recovery was defined as having no fever, as well as all of the visual analog scale scores (sore throat, cough, myalgia and fatigue) staying at 0 cm for more than 48 h.

KGLY, Kegan Liyan oral liquid; LHQW, Lianhuaqingwen capsules; FAS, full analysis set; RR, risk ratio (KGLY vs. LHQW); CI, confidence interval.

**Table 6:** Time to symptom remission in the KGLY and LHQW arms (FAS).

Time to symptom remission (days)^a^	KGLY: *n* = 61; LHQW: *n* = 60
KGLY, median (interquartile range), days	10 (8,>10)
LHQW, median (interquartile range), days	10 (7,>10)
HR (95% CI)	0.90 (0.57 to 1.43)
*p* value	0.62

^a^ Time to symptom remission was defined as the interval between the initial intervention to symptom remission in days. Symptom remission was defined as having no fever, as well as all of the visual analog scale scores (sore throat, cough, myalgia and fatigue) staying between 0 and 1 cm for more than 48 h.

KGLY, Kegan Liyan oral liquid; LHQW, Lianhuaqingwen capsules; FAS, full analysis set; HR, hazard ratio (KGLY vs. LHQW); CI, confidence interval.

**Table 7:** Recovery rates of selected symptoms in the KGLY and LHQW arms (FAS).

	Day 3	Day 7	Day 10
Sore throat^a^	KGLY: *n* = 60; LHQW: *n* = 58		
KGLY, n (%)	7 (11.7)	36 (60.0)	48 (80.0)
LHQW, n (%)	3 (5.2)	30 (51.7)	44 (75.9)
RR (95% CI)	1.70 (0.65 to 4.45)	1.19 (0.82 to 1.71)	1.13 (0.74 to 1.71)
*p* value	0.35	0.37	0.59
Fever^a^^,^^b^	KGLY: *n* = 41; LHQW: *n* = 45		
KGLY, n (%)	30 (73.2)	41 (100.0)	41 (100.0)
LHQW, n (%)	36 (80.0)	45 (100.0)	45 (100.0)
RR (95% CI)	0.83 (0.49 to 1.41)	/	/
*p* value	0.45	/	/
Cough^a^	KGLY: *n* = 60; LHQW: *n* = 58		
KGLY, n (%)	1 (1.7)	9 (15.0)	19 (31.7)
LHQW, n (%)	1 (1.7)	11 (19.0)	20 (34.5)
RR (95% CI)	0.98 (0.24 to 3.98)	0.87 (0.56 to 1.36)	0.94 (0.64 to 1.38)
*p* value	1.0000	0.57	0.75
Myalgia^a^	KGLY: *n* = 49; LHQW: *n* = 49		
KGLY, n (%)	14 (28.6)	35 (71.4)	43 (87.8)
LHQW, n (%)	19 (38.8)	38 (77.6)	46 (93.9)
RR (95% CI)	0.80 (0.54 to1.19)	0.85 (0.52 to 1.39)	0.65 (0.25 to 1.66)
*p* value	0.29	0.49	0.29
Fatigue^a^	KGLY: *n* = 54; LHQW: *n* = 55		
KGLY, n (%)	10 (18.5)	31 (57.4)	37 (68.5)
LHQW, n (%)	12 (21.8)	31 (56.4)	43 (78.2)
RR (95% CI)	0.91 (0.59 to 1.40)	1.02 (0.70 to 1.49)	0.77 (0.48 to 1.24)
*p* value	0.67	0.91	0.25

a Recovery rates of selected symptoms (including sore throat, cough, myalgia and fatigue) were defined as the proportion of subjects with a pre-treatment symptom score > 0 who achieved a post-treatment score = 0.

b Fever was defined as having subaxillary temperature ≥ 37.3°C.

KGLY, Kegan Liyan oral liquid; LHQW, Lianhuaqingwen capsules; FAS, full analysis set; RR, risk ratio (KGLY vs. LHQW); CI, confidence interval.

**Figure 4:**
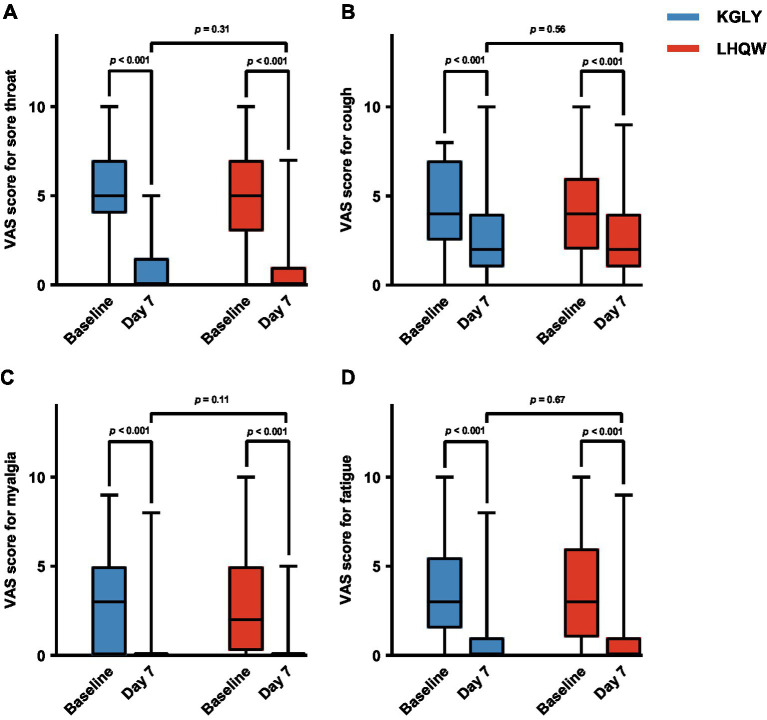
Comparison of VAS scores of selected symptoms at baseline and day 7 in the KGLY and LHQW arms: sore throat (panel **A**), cough (panel **B**), myalgia (panel **C**) and fatigue (panel **D**). In each boxplot, the central mark indicated the median, and the edges of the box referred to the 25th and 75th percentiles. The extended lines showed the full range. VAS, visual analog scale; KGLY, Kegan Liyan oral liquid; LHQW, Lianhuaqingwen capsules.

The VAS score for the selected symptoms (sore throat, cough, myalgia and fatigue) decreased significantly in both groups after treatment (*p* < 0.001), and no notable differences were identified in post-treatment VAS scores for any of the symptoms, or change of VAS scores for any of the symptoms, between groups (*p* > 0.05 for all outcomes). For the entire 10-day period, more participants in KGLY group had their symptom of sore throat completely resolved (80.0% vs. 75.9%, *p* = 0.59) and the mean AUC for sore throat VAS scores were lower in KGLY group than in LHQW group (15.23 ± 9.72 vs. 16.45 ± 10.88, difference − 1.22, *p* = 0.52), but the difference was not statistically significant. Similarly, though no notable differences were identified, decrease of sore throat VAS scores from baseline until day 10 was greater in KGLY group (−4.59 ± 2.42 cm vs. −4.03 ± 2.81 cm, difference − 0.56 cm, *p* = 0.25; Tables 6–11; Figure 4).

**Table 8:** Time to recovery of selected symptoms in the KGLY and LHQW arms (FAS).

Time to recovery of selected symptoms^a^	
Sore throat (days)	KGLY: *n* = 60; LHQW: *n* = 58
KGLY, median (interquartile range), days	7 (5,10)
LHQW, median (interquartile range), days	7 (5,10)
HR (95% CI)	1.18 (0.79 to 1.78)
*p* value	0.35
Fever (days)	KGLY: *n* = 41; LHQW: *n* = 45
KGLY, median (interquartile range), days	3 (2,4)
LHQW, median (interquartile range), days	3 (2,3)
HR (95% CI)	0.78 (0.50 to 1.20)
*p* value	0.15
Cough (days)	KGLY: *n* = 60; LHQW: *n* = 58
KGLY, median (interquartile range), days	>10 (7,>10)
LHQW, median (interquartile range), days	>10 (10,>10)
HR (95% CI)	0.88 (0.47 to 1.64)
*p* value	0.66
Myalgia (days)	KGLY: *n* = 49; LHQW: *n* = 49
KGLY, median (interquartile range), days	5 (3,10)
LHQW, median (interquartile range), days	5 (3,7)
HR (95% CI)	0.81 (0.54 to1.23)
*p* value	0.25
Fatigue (days)	KGLY: *n* = 54; LHQW: *n* = 55
KGLY, median (interquartile range), days	7 (5,>10)
LHQW, median (interquartile range), days	7 (4,10)
HR (95% CI)	0.84 (0.54 to 1.31)
*p* value	0.40

a Time to recovery of selected symptoms (including fever, sore throat, cough, myalgia and fatigue) was defined as the interval between the initial intervention to recovery of selected symptoms. Recovery of selected symptoms (including sore throat, cough, myalgia and fatigue) was defined as post-treatment score = 0. Recovery of fever was defined as post-treatment subaxillary temperature < 37.3°C.

KGLY, Kegan Liyan oral liquid; LHQW, Lianhuaqingwen capsules; FAS, full analysis set; HR, hazard ratio (KGLY vs. LHQW); CI, confidence interval.

**Table 9:** Comparison of VAS scores of selected symptoms in the KGLY and LHQW arms (FAS).

	KGLY arm (*n* = 61)			LHQW arm (*n* = 60)			*p* value^+^
	Baseline	Day 7	*p* value^*^	Baseline	Day 7	*p* value^*^	
VAS score for sore throat, mean (SD), cm	4.82 (2.38)	0.77 (1.20)	< 0.001	4.57 (2.73)	1.03 (1.62)	< 0.001	0.31
VAS score for cough, mean (SD), cm	4.48 (2.42)	2.70 (2.36)	< 0.001	4.32 (2.86)	2.45 (2.46)	< 0.001	0.56
VAS score for myalgia, mean (SD), cm	3.15 (2.96)	0.61 (1.44)	< 0.001	2.90 (2.77)	0.27 (0.78)	< 0.001	0.11
VAS score for fatigue, mean (SD), cm	3.64 (2.71)	1.10 (2.03)	< 0.001	3.62 (2.94)	0.96 (1.76)	< 0.001	0.67

+ Comparing the two groups on day 7.

*Comparing baseline and day 7. VAS, visual analog scale; KGLY, Kegan Liyan oral liquid; LHQW, Lianhuaqingwen capsules; FAS, full analysis set; SD, standard deviation.

**Table 10:** Change in VAS scores of selected symptoms in the KGLY and LHQW arms (FAS).

	Baseline to day 3	Baseline to day 7	Baseline to day 10
Change in VAS score for sore throat	KGLY: *n* = 61; LHQW: *n* = 60		
KGLY, mean (SD), cm	−1.64 (2.35)	−4.05 (2.25)	−4.59 (2.42)
LHQW, mean (SD), cm	−0.90 (3.01)	−3.53 (2.85)	−4.03 (2.81)
MD (95% CI), cm	−0.74 (−1.71 to 0.23)	−0.51 (−1.44 to 0.41)	−0.56 (−1.50 to 0.39)
*p* value	0.13	0.27	0.25
Change in VAS score for cough	KGLY: *n* = 61; LHQW: *n* = 60		
KGLY, mean (SD), cm	0.26 (2.83)	−1.77 (2.78)	−3.00 (2.42)
LHQW, mean (SD), cm	0.23 (2.47)	−1.87 (2.91)	−2.78 (2.89)
MD (95% CI), cm	0.03 (−0.93 to 0.99)	0.10 (−0.93 to 1.12)	−0.48 (−1.18 to 0.74)
*p* value	0.95	0.85	0.66
Change in VAS score for myalgia	KGLY: *n* = 61; LHQW: *n* = 60		
KGLY, mean (SD), cm	−0.93 (2.50)	−2.54 (2.55)	−2.93 (2.85)
LHQW, mean (SD), cm	−1.53 (2.50)	−2.63 (2.77)	−2.85 (2.75)
MD (95% CI), cm	0.60 (−0.30 to 1.50)	0.09 (−0.86 to 1.05)	−0.08 (−1.09 to 0.92)
*p* value	0.19	0.85	0.87
Change in VAS score for fatigue	KGLY: *n* = 61; LHQW: *n* = 60		
KGLY, mean (SD), cm	−1.05 (2.56)	−2.54 (2.57)	−2.97 (2.55)
LHQW, mean (SD), cm	−1.28 (1.93)	−2.67 (2.68)	−2.98 (2.90)
MD (95% CI), cm	0.23 (−0.58 to 1.05)	0.13 (−0.82 to 1.07)	0.02 (−0.97 to 1.00)
*p* value	0.57	0.79	0.97

VAS, visual analog scale; KGLY, Kegan Liyan oral liquid; LHQW, Lianhuaqingwen capsules; FAS, full analysis set; SD, standard deviation; MD, mean difference (KGLY vs. LHQW); CI, confidence interval.

**Table 11:** AUC of the VAS score for sore throat in the KGLY and LHQW arms (FAS).

	Baseline to day 7
AUC for sore throat VAS score	KGLY: *n* = 61; LHQW: *n* = 60
KGLY (*n* = 61), mean (SD)	15.23 (9.72)
LHQW (*n* = 60), mean (SD)	16.45 (10.88)
MD (95% CI)	−1.22 (−4.93 to 2.49)
*p* value	0.52

AUC, areas under the curve; VAS, visual analog scale; KGLY, Kegan Liyan oral liquid; LHQW, Lianhuaqingwen capsules; FAS, full analysis set; SD, standard deviation; MD, mean difference (KGLY vs. LHQW); CI, confidence interval.

**Table 12:** SARS-CoV-2 RNA negative conversion in the KGLY and LHQW arms (FAS).

	Day 7	Day 10
SARS-CoV-2 RNA negative conversion^a^	KGLY: *n* = 61; LHQW: *n* = 60	
KGLY (*n* = 61), n (%)	12 (19.7)	34 (55.7)
LHQW (*n* = 60), n (%)	12 (20.0)	35 (58.3)
RR (95% CI)	0.99 (0.63 to 1.55)	0.95 (0.66 to 1.37)
*p* value	0.96	0.77

a According to the guideline on diagnosis and treatment of COVID-19 (Trial 9th edition), SARS-CoV-2 RNA negative conversion referred to two consecutive SARS-CoV-2 RNA detection tests with negative results (at least 24 h between sampling times).

KGLY, Kegan Liyan oral liquid; LHQW, Lianhuaqingwen capsules; FAS, full analysis set; RR, risk ratio (KGLY vs. LHQW); CI, confidence interval.

**Table 13:** Positive test results after negative conversion in the KGLY and LHQW arms (FAS).

	Day 10
Positive test results after negative conversion^a^	
KGLY (*n* = 12), n (%)	0 (0.0)
LHQW (*n* = 12), n (%)	2 (16.7)
RR (95% CI)	/
*p* value	0.46

a Negative conversion referred to two consecutive SARS-CoV-2 RNA detection tests with negative results (at least 24 h between sampling times).

KGLY, Kegan Liyan oral liquid; LHQW, Lianhuaqingwen capsules; FAS, full analysis set; RR, risk ratio; CI, confidence interval.

**Table 14:** The incidence of pneumonia in the KGLY and LHQW arms (FAS).

	Day 10
Incidence of pneumonia after treatment initiation	
KGLY (*n* = 61), n (%)	2 (3.3)
LHQW (*n* = 60), n (%)	0 (0.0)
RR (95% CI)	/
*p* value	0.48

KGLY, Kegan Liyan oral liquid; LHQW, Lianhuaqingwen capsules; FAS, full analysis set; RR, risk ratio; CI, confidence interval.

**Table 15:** Probability of experiencing any adverse event in the KGLY and LHQW arms (SS).

	Day 10
Probability of experiencing any adverse event	
KGLY (*n* = 63), n (%)	8 (12.7)
LHQW (*n* = 64), n (%)	13 (20.3)
RR (95% CI)	0.77 (0.53 to 1.15)
*p* value	0.25

KGLY, Kegan Liyan oral liquid; LHQW, Lianhuaqingwen capsules; SS, safety set; RR, risk ratio; CI, confidence interval.

**Table 16:** Comparison of different adverse events in the KGLY and LHQW arms (SS).

Adverse event	KGLY arm (*n* = 63)	LHQW arm (*n* = 64)
	No. of cases	No. of cases
Diarrhea	2	5
Nausea	1	3
Elevated blood platelet count	1	3
Dysmenorrhea	2	1
Pneumonia	2	0
Bacterial respiratory tract infection	0	1
Abdominal pain	1	0
Elevated alanine aminotransferase level	1	0
Elevated white blood cell count	0	1
Waist pain	0	1

KGLY, Kegan Liyan oral liquid; LHQW, Lianhuaqingwen capsules; SS, safety set.

There was no statistically significant difference between the two groups in terms of SARS-CoV-2 RNA negative conversion at day 7 (19.7% vs. 20.0%, *p* = 0.96) and day 10 (55.7% vs. 58.3%, *p* = 0.99; Table 12). Simultaneously, there were no patients in KGLY group who had a positive SARS-CoV-2 RNA test result after negative conversion, less than those in LHQW group (0% vs. 16.7%, *p* = 0.46), though not statistically significant (Table 13). The incidence of pneumonia between groups was not statistically different either (3.3% vs. 0.0%, *p* = 0.48; Table 14).

### Safety

All 127 enrolled patients were included in the SS analysis. Twenty-one individuals (8 [12.7%] in KGLY group, and 13 [20.3%] in LHQW group) reported a toal of 25 adverse events (10 in KGLY group, and 15 in LHQW group). There was no significant difference between the groups in the occurrence of adverse events (*p* = 0.25). Diarrhea, nausea, and elevated blood platelet count were the most frequent adverse events in two both arms, but only mild events were observed. No serious adverse events were reported (Tables 15, 16).

## Discussion

The present study was launched before the severe new outbreak of COVID-19 in China in late 2022, when most people had not yet contracted COVID-19, with the aim to provide more evidence for the application of TCM for COVID-19 in the future. It is to our knowledge the first randomized, double-blinded trial that compares the efficacy of KGLY with LHQW for mild COVID-19. We found that KGLY was non-inferior to LHQW in relieving fever, sore throat, cough, fatigue, and myalgia. The incidence of adverse events did not differ significantly, indicating that KGLY represents a potential therapy with a good safety profile for mild COVID-19. Therefore, taking it into account that COVID-19 is still circulating worldwide and a large number of people are experiencing repeated infections, the present trial provides a feasible option for COVID-19 treatment.

Although the post-treatment VAS scores for symptoms remarkably decreased in both groups, less patients met the criteria for symptom remission at day 10 than we expected, 58.3% in the LHQW group in 60.7% in the KGLY group. The recovery rate of cough was the lowest among the five symptoms, 34.5% in LHQW and 31.7% in KGLY. Our finding was consistent with that of Tenforde et al. (17), who found that COVID-19 can cause prolonged sickness even in people with mild infection. Among 274 symptomatic COVID-19 infected adults in a survey conducted in the United States, 35% of individuals had not returned to a normal state of health after 2–3 weeks of infection (17). Cough is one of the common symptoms in people with ‘Long COVID’ and the least likely of the symptoms to resolve (17, 18). Almost half (43%) of the patients still suffered from cough 2–3 weeks after infection, according to the above-mentioned survey (17). Besides, our study was conducted during the first large-scale wave in China since the original outbreak, when an immune barrier had not yet been established, which might be another reason why symptoms sustained for a long time.

Currently, oral antiviral agents available in China for COVID-19 include paxlovid, azvudine and molnupiravir (9). Nevertheless, these therapies are not indicated for treatment in patients with mild symptoms and no risk factors for progression to severe disease. Meanwhile, some antiviral agents are relatively expensive and source-limited during outbreaks. However, early intervention can accelerate the recovery of COVID-19 (19). Therefore, TCM provides new option for clinical management of patients with mild COVID-19.

TCM has been widely used to treat pandemics for more than 2,500 years, the first mentioning being in *Huangdi Neijing*. To date, plenty of Chinese herbs have been reported to possess antiviral activities against various coronaviruses, notably the severe acute respiratory syndrome coronavirus (SARS-CoV) and the Middle East respiratory syndrome coronavirus (MERS-CoV) (20). Due to its long history of application and demonstrated efficacy, TCM has also been recommended in COVID-19 guidelines (21). In March 2022, the WHO Expert Meeting concluded that TCM may help to shorten the time for viral clearance, promote the recovery of clinical symptoms, and reduce the risk of progressing to severe disease (22). Results from systematic reviews and meta-analyses also demonstrate that TCM treatment may promote cure and reduce clinical deterioration in patients with COVID-19 (19, 23, 24).

However, researchers have also pointed out that because of the complex situation during the epidemic, many studies on COVID-19 had severe limitations in study design, such as the lack of allocation concealment and blinding procedures, which enhances difficulties in developing clear conclusions (25). In this study, the effects of KGLY and LHQW on symptoms, viral clearance and disease progression among mild COVID-19 patients were compared head-to-head for the first time. We adopted a randomized, double-blinded and double-dummy design, consequently reducing the risk of bias and enhancing the reliability of the study.

KGLY, mainly used to clear heat and resolve dampness, originates from classic formulas Yin-Qiao-San and Shen-Jie-San, and has been used extensively for the treatment of virus infections. Yin-Qiao-San, first presented by Ju-Tong Wu in 1798, was frequently used to treat influenza (26). Shen-Jie-San, initially documented in *Shanghan Wenyi Tiaobian* in 1784, was applied for infectious diseases (27). According to the latest epidemiological investigations, *dampness* and *heat* are the main TCM pathogenic factors in people infected with Omicron variant (28, 29). Clearing heat and resolving dampness are the first priority when dealing with *dampness* and *heat* in COVID-19 according to the eminent TCM expert Zhongying Zhou (30). Fever (83.0% of the cases), sore throat (62.1%), cough (89.7%), fatigue (84.1%), and myalgia (72.7%) were the most common symptoms after infection reported by a cross-sectional study that surveyed 630 Omicron-infected patients (1). In TCM, fever and sore throat are among the signs of *Dampness-heat in the Lung* syndrome. The above features of Omicron infection fit with the indications of KGLY well.

Pharmacological basis for understanding the effects of KGLY for COVID-19 has been established through research. A quality check of KGLY by high-performance liquid chromatography technique identified 9 compounds, including chlorogenic acid, 4’-O-beta-glucopyranosyl-5-O-methylvisamminol, ammonium glycyrrhizinate, geniposide, forsythin, caffeic acid, baicalin, tectoridin, and menthol (31). Among them, baicalin possesses significant antiviral effects against SARS-CoV-2 by altering respiratory microbiome and ameliorating the cytokine storm through TNF and IL-17 pathways (32–34). Geniposide suppresses virus replication *in vitro* via modifying Ca^2+^ signaling pathway (35). Chlorogenic acid is effective against coronavirus infection by targeting apoptosis, particularly impacting the initial stage of virus replication and release (36). And caffeic acid derivatives have shown strong interactions with SARS-CoV-2 proteins in molecular docking simulations (37). Meanwhile, previous studies have demonstrated that KGLY possessed anti-coronavirus function both *in vivo* and *in vitro* (38, 39). Moreover, KGLY was reported to decrease inflammatory factors IL-1β, IL-6, and TNF-*α* (31) and could treat influenza virus infection by lowering the oxidative stress level through increased superoxide dismutase activity (40).

This study had several limitations. First, stringent COVID-19 prevention and control measures in China precluded launching a multicenter study, so this trial was carried out at two centers with a relatively small sample size. As a result, the recruitment was limited to a certain geographic region, which may impact the generalizability of the findings. Second, the follow-up was relatively brief, which failed to provide further evidence for subsequent efficacy. It is unclear whether the symptoms will rebound afterwards. Finally, different strains of COVID-19 have diverse characteristics, thus, sequencing data would be conducive to instructing precise treatment of the strain. According to the epidemic situation then, Omicron was the main strain during the study period. However, strains were not sequenced in the present trial, requiring further studies.

## Conclusion

Based on the established efficacy of LHQW in treating COVID-19, our findings demonstrate that KGLY is non-inferior in terms of symptom remission. This non-inferiority is significant as it positions KGLY as a potentially effective alternative treatment option. However, further studies are warranted to confirm the broader efficacy and safety of KGLY for COVID-19 management.

## Data Availability

The raw data supporting the conclusions of this article will be made available by the authors, without undue reservation.

## Glossary

AUC: area under the curve
CI: confidence interval
CONSORT: Consolidated Standards of Reporting Trials
COVID-19: Coronavirus disease 2019
FAS: full analysis set
GCP: Good Clinical Practice
HPLC: high performance liquid chromatography
HR: hazard ratio
IQR: interquartile range
KGLY: Kegan Liyan oral liquid
KUMCR: Key Unit of Methodology in Clinical Research
LHQW: Lianhuaqingwen capsule
MD: mean difference
MERS-CoV: Middle East respiratory syndrome coronavirus
p.p.: percentage point
PPS: per protocol set
RR: risk ratio
SARS-CoV: severe acute respiratory syndrome coronavirus
SARS-CoV-2: severe acute respiratory syndrome coronavirus 2
SD: standard deviation
SS: safety set
TCM: traditional Chinese medicine
ULN: upper limit of normal
VAS: visual analog scale
